# In Search of Small Molecules That Selectively Inhibit MBOAT4

**DOI:** 10.3390/molecules26247599

**Published:** 2021-12-15

**Authors:** Emily S. Murzinski, Ishika Saha, Hui Ding, David Strugatsky, Ryan A. Hollibaugh, Haixia Liu, Peter Tontonoz, Patrick G. Harran

**Affiliations:** 1 Department of Chemistry and Biochemistry, University of California, Los Angeles, CA 90024, USA; esmurzinski@gmail.com (E.S.M.); ishikasaha@ucla.edu (I.S.); huiding@chem.ucla.edu (H.D.); ryanhollibaugh@gmail.com (R.A.H.); haixia.liu@roche.com (H.L.); 2 Department of Microbiology, Immunology & Molecular Genetics, California and Nano Systems Institute, University of California, Los Angeles, CA 90024, USA; strugd@g.ucla.edu; 3 Department of Biological Chemistry, University of California, Los Angeles, CA 90024, USA; PTontonoz@mednet.ucla.edu

**Keywords:** ghrelin *O*-acyl transferase, MBOAT 4, peptidomimetic

## Abstract

Ghrelin is a 28-residue peptide hormone produced by stomach P/D1 cells located in oxyntic glands of the fundus mucosa. Post-translational octanoylation of its Ser-3 residue, catalyzed by MBOAT4 (aka ghrelin *O*-acyl transferase (GOAT)), is essential for the binding of the hormone to its receptor in target tissues. Physiological roles of acyl ghrelin include the regulation of food intake, growth hormone secretion from the pituitary, and inhibition of insulin secretion from the pancreas. Here, we describe a medicinal chemistry campaign that led to the identification of small lipopeptidomimetics that inhibit GOAT in vitro. These molecules compete directly for substrate binding. We further describe the synthesis of heterocyclic inhibitors that compete at the acyl coenzyme A binding site.

## 1. Introduction

**Figure 1:**
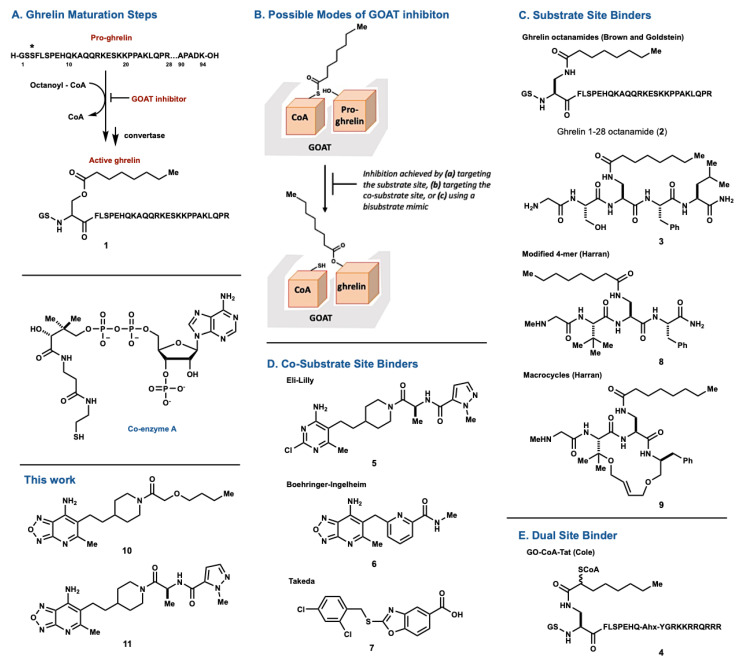
(**A**) GOAT-catalyzed octanoylation of pro-ghrelin is a critical part of ghrelin maturation. GOAT is weakly inhibited by its reaction product, acyl ghrelin (**1**), and more potently by analog **2**, wherein Ser-3 is replaced by diaminopropionic acid (DAP). Systemic inhibition of GOAT is expected to reduce circulating acyl-ghrelin levels, leading to improved insulin response to glucose challenge. (**B**) GOAT inhibition can be achieved by targeting either the substrate or co-substrate site, or by targeting both sites with a “bisubstrate” mimic. (**C**–**E**) Known inhibitors of GOAT.

Ghrelin is a 28-residue lipopeptide (Figure 1A) discovered by Kojima and co-workers as the endogenous ligand for the growth hormone secretagogue receptor (GHS-r) [1]. It was found that the octanoylation of Ser3 was required for ghrelin’s endocrine activities. At the time, the enzyme responsible for this modification was unknown. Later, Yang hypothesized this atypical lipidation was likely performed by a member of the membrane-bound *O*-acyl transferase (MBOAT) family of enzymes [2]. The MBOATs catalyze the lipidation of a variety of substrates including phospholipids, neutral lipids, and proteins using saturated and unsaturated acyl coenzyme-A derivatives as acyl donors [3]. In 2008, Yang et al. utilized a candidate cloning approach to demonstrate that MBOAT 4, now termed ghrelin *O*-acyl transferase (GOAT), the only MBOAT capable of catalyzing proghrelin octanoylation [2]. Gutierrez used a candidate gene silencing approach to independently reach the same conclusion for human GOAT [4]. Short-interfering RNAs (siRNAs) were produced and the effects of silencing individual MBOAT genes were observed. MS/MS fragmentation analyses of GOAT acylated ghrelin showed that the acylation is on serine-3, identical to acyl ghrelin produced in the stomach.

Active ghrelin (hereafter referred to as ghrelin) was shown to induce adiposity in rodents and food intake in both rodents and humans [5,6]. Ghrelin was also found to play a role in glucose homeostasis by inhibiting insulin secretion from the pancreas [7,8]. Levels of active ghrelin in the blood peak during fasting and decrease after meals [9,10]. Ghrelin is primarily produced by peripheral tissues, rather than the central nervous system [11]. In addition, no other hormone is known to be octanoylated [12]. Given the biological specificity of GOAT and ghrelin, it was thought that, unlike GHS-r ligands, an inhibitor of GOAT would not need to penetrate the brain to cause pharmacological effects. The potential therapeutic effect of suppressing circulating ghrelin levels fueled efforts to identify inhibitors of the enzyme. Such molecules could clarify the roles of ghrelin in feeding, weight gain and glucose homeostasis, both in normal physiology and in disease.

A general model of GOAT transmembrane architecture was proposed, which describes eleven membrane-spanning helix domains and two intramembrane domains [13,14]. Although integral membrane proteins constitute an essential portion of the proteome, they are often difficult to purify and analyze because of their hydrophobicity and reliance on lipid–bilayer interactions for stability and activity. Since a purified form of GOAT has yet to be described, medicinal chemistry efforts lack the benefit of high-resolution structural data and detailed binding models. Crude membrane preparations from GOAT expressing cells are used as a source of enzymatic activity for inhibitor assays. Despite this challenge, several groups reported GOAT inhibitors (Figure 1B–E) [15,16,17]. Shortly after identifying GOAT, Yang et al. demonstrated the enzyme was inhibited by its reaction product **1**, and more potently by esterase resistant octanamide analog **2** (IC_50_ = 200 nM) [18]. A truncated peptide (**3**) consisting of the first five N-terminal ghrelin residues also inhibited GOAT, albeit five-fold less potently (IC_50_ = 0.1 µM). Barnett et al. developed ‘bi-substrate’ mimic GO-CoA-Tat (**4**, IC_50_ = 3 mM). This inhibitor incorporated the first ten N-terminal residues of active ghrelin, which is S-linked to a coenzyme A molecule, and fused to a Tat sequence to promote endocytosis [19]. We found GO-CoA-Tat less potent than **2** in vitro, but it was reported to reduce circulating ghrelin levels in vivo, improve glucose tolerance and limit weight gain in mice fed a high-fat diet. These findings were attributed to effects on ghrelin signaling because they were not observed in ghrelin knockout mice. Eli Lilly, Boehringer Ingelheim and Takeda recently patented more drug-like, heterocyclic inhibitors of GOAT [20,21,22]. Takeda showed that their inhibitor **7** competed at the CoA site on GOAT, presumably by mimicking elements of an adenine base. It is likely that amino pyrimidine **5** and oxadiazolopyridine **6** function similarly.

Whereas GO-CoA-Tat exploits the ability of a polyarginine sequence to promote active cellular uptake, we sought a ghrelin peptidomimetic showing passive membrane permeability. There was precedent for this outcome; for example, the marketed drug Aliskiren was designed based on the peptide sequence of renin [23]. Nonetheless, we faced a difficult problem. Rather than elaborating a screening hit to a (typically larger) end-product, an effective GOAT inhibitor would need to be smaller, less polar, and metabolically stable relative to the parent peptide. We sought to identify and enhance key binding interactions while removing superfluous functionality. Herein, we report peptidomimetic GOAT inhibitors, including **8** and **9,** and describe their in vitro performance. We also outline the synthesis of novel hybrid heterocyclic GOAT inhibitors based on the structures of prototypes **5** and **6**.

## 2. Results and Discussion

**Figure 2:**
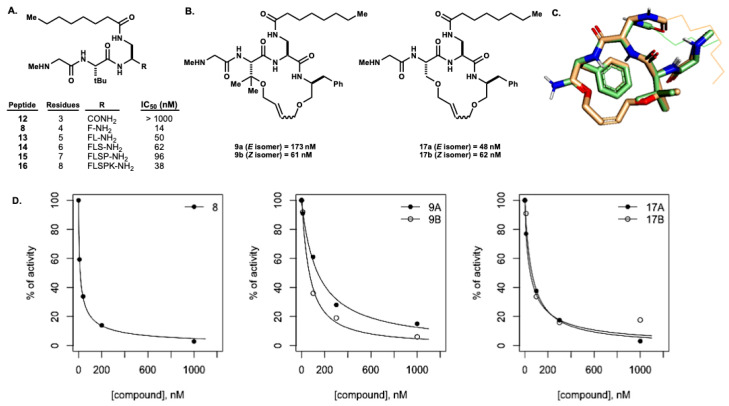
(**A**) Ghrelin truncation peptide series and respective IC_50_ values. Enzyme activity was quantified using the method described by Yang et al. [18] (see Materials and Methods); (**B**) Macrocycles derived from the parent peptides and respective IC_50_ values; (**C**) Structural overlay of **9b** (orange) against **8** (green), octanamide chains are indicated as line bonds for clarity; (**D**) In vitro inhibition of proghrelin octanoylation mediated by GOAT using the shown compounds. The experimental data were fit to a 4-parameter logistic model, (Bottom − Top)/(1 + (I/IC_50_)^n^) + Top [24] using DYNAFIT software [25]. The Top was defined as reaction conditions without inhibitor and the Bottom was defined as reaction conditions with full inhibition by 5 μM concentration of **2**. Top and Bottom conditions were included in every experiment.

Building on data reported by Brown and Goldstein [18], we began our studies by evaluating a series of modified truncation peptides ranging from 3–8 amino acids in length (Figure 2). We discovered that mono methylating the N-terminus and replacing Ser2 with t-butyl glycine increased the in vitro potency of 5-mer **3** by 20-fold (see **13**, Figure 2). The corresponding 4-mer **8** was even more effective. It showed an IC_50_ of 14 nM when assayed in membrane fractions. The phenyl ring in **8** was important. Analogs of **8** with either histidine or tyrosine at P4 were inactive, as was three residue peptide **12**.

Peptides chains longer than three amino acids are generally not taken up into cells passively. However, their cyclic counterparts can show an improved stability, pharmacokinetics and, if their structure facilitates internal shielding of the polar surface area, useful levels of cell permeability [26]. We used Schrödinger’s Macromodel software to conduct molecular modeling studies, using AMBER in a continuum solvation model (octanol) to inform the design of hypothetical macrocyclic analogs of compound **8**, which was our best performing tetrapeptide. During these studies, we were pleased to find that the peptide backbone of the macrocyclic analog **9b** overlaid almost perfectly with that of **8** (Figure 2C)**.** The root mean square deviation (RMSD) between the two structures was 0.06 Å at the α-carbon atoms for the lowest energy conformations of both molecules. We ignored variations of the octanamide chain in these analyses as this group was expected to be highly flexible. Based on our observations, we synthesized cyclic variants **9** and **17** using catalyzed ring-closing metatheses of bis-allyl ether precursors, (Figure 2), resulting in mixtures of separable alkene geometric isomers. The ability of these compounds to inhibit GOAT activity in vitro was comparable to their linear peptide counterparts. Moreover, enzymatic reaction rates measured while varying substrate concentrations in the presence of a fixed concentration of **17a** suggested that the compound inhibited GOAT by competing for the peptide substrate (see Materials and Methods). With both linear and cyclic inhibitors in hand, we began an extensive program to synthesize and assay analogs, wherein the octanamide side chain, the P4 aromatic moiety, N-methylation patterns, ring substituents and connectivity (for **9**/**17**) and stereochemistry were varied in search of molecules that inhibited GOAT potently in vitro, which could block acyl ghrelin secretion from an engineered insulinoma cell line (INS-1, see Materials and Methods). Over time hundreds of compounds were evaluated [27,28]. While several of those components inhibited GOAT activity in membrane fractions in the 50–200 nM range, none were superior to prototypic peptide **8** and none, including **8**, showed significant activity in cell culture. Actually, the best performing oligomer in cell culture was lipopentapeptide **13** (Figure 2). Unfortunately, that molecule showed poor pharmacokinetics and a low oral bioavailability in mice.

During this time, pharmaceutical companies began publishing patent literature on GOAT inhibitors discovered through high-throughput screening. Takeda reported data indicating a series of benzoxazole carboxylates (including **7** in Figure 1D), which inhibited the enzyme by competing at its co-enzyme A binding site [21]. Based on their structures, we believed it likely the aminopyrimidines reported by Eli Lilly [20] and the oxadiazolopyridines described by Boehringer Ingelheim [22] functioned similarly. Because numerous metabolic enzymes utilize co-enzyme A derivatives for catalysis, there was the risk of the unanticipated off-target effects for inhibitors of this kind. Nonetheless, Eli Lilly reported that compound **5** was orally bioavailable and inhibited ghrelin production in vivo at doses that did not cause observable adverse side effects.

We sought a hybrid molecule that would contain an oxadiazolopyridine linked to an octanoyl motif through the piperidinyl ethyl scaffold used in Eli Lilly compound **5** (and also a feature of certain ACAT inhibitors) [29]. We first synthesized compounds **5** and **6** in house and tested them in our own assays. We found the membrane fraction IC_50_ = 88 nM and 64 nM, as well as INS-1 cellular IC_50_ = 670 nM and 540 nM, respectively. Having roughly confirmed literature activities, we targeted new hybrids **10** and **11** wherein the oxadiazolopyridine would replace the aminopyrimidine in **5** and, in **10**, its dipeptidyl segment would be replaced by an octanoyl unit, a feature essential for activity in our previous peptidomimetic efforts.

**Scheme 1:**
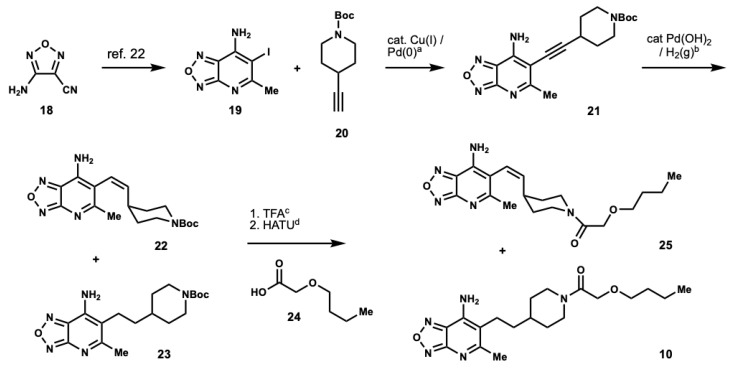
**18** was prepared using the procedure described in ref. 30. (**a**) **19** (1 eq.), **20** (1.5 eq), CuI (0.1 eq), (PPh_3_)_2_PdCl_2_ (0.2 eq), Et_3_N (0.25 M), 80 °C, 51%; (**b**) Pd(OH)_2_ (20% on carbon) (0.3 eq.), EtOH (0.1 M), H_2_ atm.; (**c**) CH_2_Cl_2_ (0.04 M), trifluoroacetic acid (TFA) (10% *v*/*v*); (**d**) **24** (1.1 eq), HATU (1.1 eq.), DIPEA (3 eq.), DMF (0.2 M), 32% **25** over three steps, 1% **10** over three steps.

Amino-iodinated oxadiazolopyridine **19** (Scheme 1) was synthesized from amino cyano oxadiazole **18** and ethyl acetoacetate, as described by Boehringer Ingelheim. Sonogashira coupling of **19** with 4-ethynyl piperidine **20** (prepared via Ohira–Bestmann homologation of the corresponding aldehyde) gave chromophore **21**. The attempted saturation of the alkyne in this molecule by hydrogenation over PtO_2_ gave mainly *cis*-alkene **22** alongside a by-product that lacked an oxadiazole ring, presumably formed via N-O bond reduction. The attempted hydrogenation over various other heterogeneous and homogeneous catalysts gave similar results. Only when catalytic Pd(OH)_2_/C was used (EtOH, 1 atm H_2_(g)), were small amounts of alkane **23** formed in a mixture that predominately contained **22**. Treatment of the crude mixture with TFA followed by acylation with butylated glycolic acid **24** allowed pure target **10** to be isolated (along with alkene congener **25**) following column chromatography on silica gel, albeit in low yield.

**Scheme 2:**
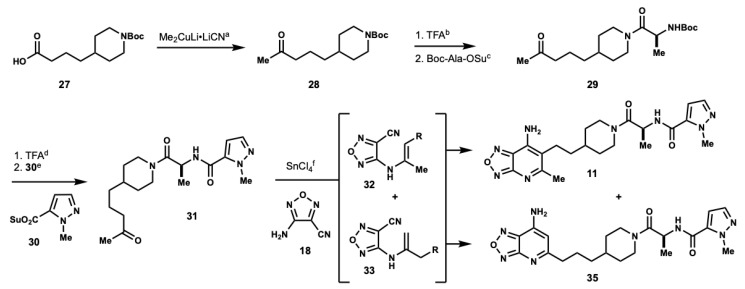
(a) CuCN (5 eq.), Et_2_O (0.6 M), MeLi (1.6M in Et_2_O; 10 eq.), 0 °C; **27** (1 eq.), Et_2_O (0.2 M), 71%; (b) CH_2_Cl_2_ (0.07 M), trifluoroacetic acid (TFA) (10% *v*/*v*), 0 °C; (c) Boc-Ala-OSu (1.1 eq.), DIPEA (3.1 eq.), MeCN (0.2 M), 0 °C, 50% over two steps; (d) CH_2_Cl_2_ (0.07 M), trifluoroacetic acid (TFA) (10% *v*/*v*), 0 °C; (e) **30** (1 eq.), DIPEA (3 eq.), MeCN (0.2 M), 0 °C, 72% over two steps; (f) **18** (1 eq.), SnCl_4_ (2 eq.), toluene (0.2 M), reflux.

To avoid the problematic hydrogenation of **21**, we developed a different route to access target **11**, a path also applicable to **10**. In this sequence, the oxadiazolopyridine unit is installed late via annulation onto an intermediate already at the desired oxidation state. We required a protected 5-piperidinyl-2-pentanone for this purpose. Boc derivative **28** (Scheme 2) was known, although it was prepared via a 3-step sequence beginning with a relatively costly starting material [30]. We instead developed a one-step synthesis of **28** from commercial acid **27** using Me_2_CuLi.LiCN, as described by Posner and Genna [31]. Multiple grams of **28** were prepared readily using this method. Degradation of the carbamate in **28** with TFA and acylation of the incipient amino ketone salt with Boc-L-Ala-OSu produced piperidyl amide **29**. Further N-terminal extension with N-Me pyrazole derivative **30** afforded compound **31**. Structure **35** contains the dipeptide segment of Eli Lilly compound **5** tethered to a methyl ketone handle from which varied heterocycles can derive. To prepare target oxadiazolopyridine **11**, we condensed **31** with cyanofurazan **18 [32]** using SnCl_4_ as promoter. This construction was originally developed by Vasil’ev et al. [33] and, in the current example, affords oxadiazolopyridine **11**, directly following aqueous workup. A second regioisomer (**35**) was also isolated from the reaction (dr = 1.5:1), a result we interpret in terms of the competing formation of regioisomeric enamine intermediates **32**/**33** that cyclize onto the pendant nitrile. Subsequent tautomerization would afford oxadiazolopyridines.

**Figure 3:**
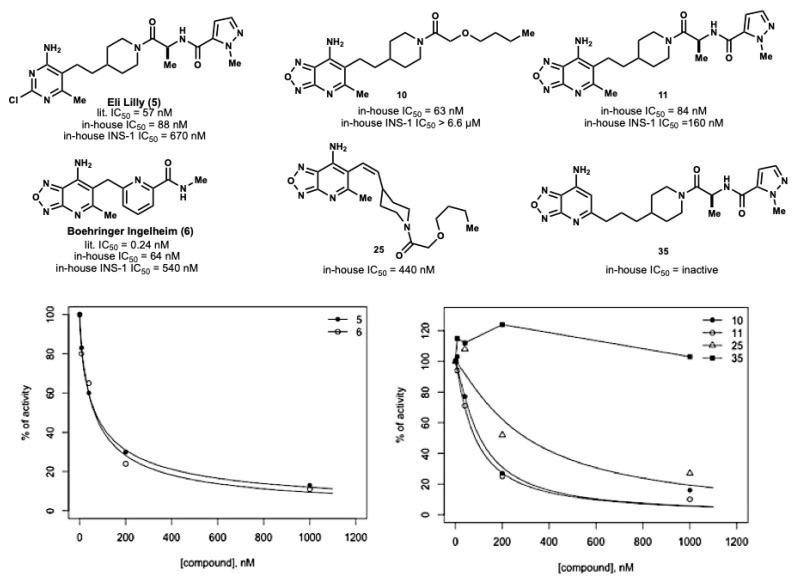
*(***top***)* In vitro and cellular activities of heterocyclic analogues (see Materials and Methods for assay descriptions); *(***bottom***)* In vitro inhibition of proghrelin octanoylation mediated by GOAT using the shown compounds. The experimental data was fit to a 4-parameter logistic model, (Bottom − Top)/(1 + (I/IC_50_)^n^) + Top [24] using DYNAFIT software [25]. The Top was defined as reaction conditions without inhibitor and the Bottom was defined as reaction conditions with full inhibition by 5 μM concentration of **2**. Top and Bottom conditions were included in every experiment.

Synthetic compounds **10**, **11**, alkene **25** and regiosiomer **35** were tested in membrane fractions containing GOAT and in ghrelin-secreting INS-1 cells. As shown in Figure 3, both **25** and **35** were significantly impaired, likely a result of their altered geometries. However, both **10** and **11** inhibited membrane GOAT as potently as did **5** and **6**. Notably, compound **11** blocked ghrelin secretion from INS-1 cells 4-fold more potently than **5**. To date, it is the most potent compound we have tested in our cellular assay. Because **10** is much less active in cells, yet it inhibits GOAT in membrane fractions just as well as **11**, we speculate that the adenine mimcry provided by the oxadiazolopyridine is key, with a linked functionality contributing mainly to stability and cellular uptake, or lack thereof.

## 3. Materials and Methods

NMR spectra were recorded on Bruker Advance spectrometers (300 MHz, 400 MHz, 500 MHz) and are reported as δ values in ppm relative to CDCl_3_ (calibrated to 7.26 ppm in ^1^H NMR and 77.16 ppm in ^13^C NMR, unless otherwise indicated). Splitting patterns are abbreviated as follows: singlet (s), doublet (d), triplet (t), quartet (q), multiplet (m), broad (br), and combinations thereof. Column chromatography was conducted on silica gel 60 (240–400 mesh) purchased from Sillicycle. Thin-layer chromatography (TLC) was performed using pre-coated, glass-backed plates (silica gel 60 PF254, 0.25 mm) and visualized using a combination of UV and potassium permanganate staining. HPLC analyses were carried out using an Agilent 1200 HPLC system equipped with an Agilent Quadrupole 6130 ESI-MS detector. Mobile phase was prepared with 0.1% TFA.

### 3.1. Preparation of Macrocycles ***9A*** and ***9B***

**Scheme 3:**
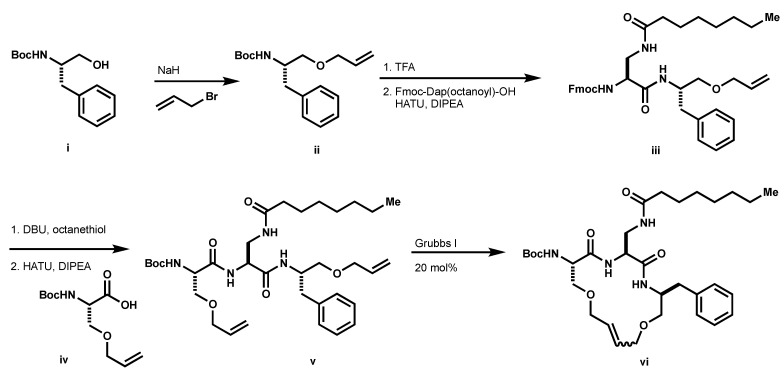
Synthetic route towards **9A** and **9B** (see Materials and Methods for experimental detail).

*tert-butyl (S)-(1-(allyloxy)-3-phenylpropan-2-yl)carbamate* (**ii, Scheme 3**): To a solution of **i** (1.0 g, 3.98 mmol) in DMF (22 mL) at 0 °C, NaH was added (60% in mineral oil, 365 mg, 9.12 mmol) followed by allyl bromide (0.77 mL, 9.72 mmol). The resulting mixture was stirred at room temperature for 4 h. The reaction was quenched with saturated aqueous NH_4_Cl and extracted with EtOAc (×2). The organics were dried (MgSO_4_), filtered, and concentrated under reduced pressure. The resultant oil was purified by column chromatography using 9:1 hexanes: EtOAc as eluents to afford **ii** (526 mg, 45%) as a clear oil. ^1^H NMR (500 MHz, CDCl_3_) δ 7.28 (q, *J* = 4.9 Hz, 2H), 7.21 (d, *J* = 7.4 Hz, 3H), 5.91 (m, 1H), 5.27 (q, *J* = 6.3 Hz, 1H), 5.19 (q, *J* = 4.0 Hz, 1H), 4.86 (s, 1H), 3.96 (m, 2H), 3.34 (m, 2H), 2.87 (m, 2H), 1.42 (s, 9H). HPLC/MS MH^+^ 192.1 (-Boc).

*(9H-fluoren-9-yl) methyl((S)-1-(((S)-1-(allyloxy)-3-phenylpropan-2-yl)amino)-3-octanamido-1-oxopropan-2-yl)carbamate* (**iii**): To a solution of **ii** (1 eq.) in CH_2_Cl_2_ (0.4 M) was added TFA (10% *v*/*v*). The resulting solution was stirred at room temperature until completion as determine by LCMS. The reaction mixture was concentrated under reduced pressure and the resultant TFA salt was used without further purification. The resultant residue (1.1 eq.), Fmoc-Dap(octanoyl)-OH (1 eq.) and HATU (1.1 eq.) was suspended in DMF (0.3 M). To the suspension DIPEA was added (2.5 eq.) and the resulting solution was stirred at room temperature until completion, as determined by LCMS. Upon completion, the reaction mixture was diluted with EtOAc and washed with saturated aqueous NH_4_Cl (×2), water (×3), saturated aqueous NaHCO_3_ (×1), and brine (×1). The organics were dried over MgSO_4_, filtered, and concentrated under reduced pressure to afford **iii** (178 mg, 95%). ^1^H NMR (500 MHz, CDCl_3_) δ 7.77 (d, *J* = 7.5 Hz, 2H), 7.60 (d, *J* = 7.2 Hz, 2H), 7.40 (t, *J* = 7.5 Hz, 2H), 7.32 (m, 2H), 7.25 (d, *J* = 14.7 Hz, 2H), 7.19 (q, *J* = 3.5 Hz, 3H), 6.97 (d, *J* = 7.8 Hz, 1H), 6.41 (d, *J* = 4.3 Hz, 1H), 6.12 (s, 1H), 5.84 (m, 1H), 5.22 (d, *J* = 17.1 Hz, 1H), 5.12 (d, *J* = 10.3 Hz, 1H), 4.37 (t, *J* = 8.9 Hz, 1H), 4.31 (m, 2H), 4.21 (t, *J* = 7.1 Hz, 2H), 3.94 (s, 2H), 3.69 (d, *J* = 11.6 Hz, 1H), 3.49 (t, *J* = 7.0 Hz, 1H), 3.37 (s, 2H), 2.87 (m, 2H), 2.13 (t, *J* = 7.2 Hz, 2H), 1.74 (s, 1H), 1.60 (s, 2H), 1.26 (t, *J* = 5.2 Hz, 8H), 0.85 (t, *J* = 7.0 Hz, 3H). ^13^C NMR (126 MHz, CDCl_3_) δ 175.37, 169.47, 143.74, 141.29 (d, *J* = 2.4 Hz), 137.81, 134.40, 129.30, 128.44, 127.80, 127.15 (d, *J* = 2.7 Hz), 126.53, 125.20, 120.03, 117.20, 72.14, 69.90, 67.52, 56.73, 50.35, 47.07, 42.15, 37.54, 36.51, 31.67, 29.24, 29.01, 25.66, 22.62, 14.06. HPLC/MS MH^+^ 626.1.

*Boc-Ser(O-allyl)-OMe*: To a solution of Boc-Ser-OMe (1.28 g, 5.85 mmol) in THF (11.7 mL) a solution of allyl ethyl carbonate (1.54 mL, 11.7 mmol), allyl palladium chloride dimer (43 mg, 0.12 mmol) and PPh_3_ (138 mg, 0.53 mmol) was added dropwise to THF (5.85 mL). The resulting reaction mixture was refluxed under argon overnight. The reaction mixture was concentrated under a reduced pressure and the resultant residue was purified by column chromatography using 9:1 hexanes: EtOAc as eluents to afford the desired product (1.24 g, 82%) as a clear oil. ^1^H NMR (500 MHz, CDCl_3_) δ 5.82 (m, 1H), 5.37 (d, *J* = 8.1 Hz, 1H), 5.23 (m, 1H), 5.17 (d, *J* = 10.4 Hz, 1H), 4.42 (t, *J* = 4.4 Hz, 1H), 3.97 (t, *J* = 5.7 Hz, 2H), 3.84 (q, *J* = 4.1 Hz, 1H), 3.75 (s, 9H), 3.64 (q, *J* = 4.2 Hz, 1H). ^13^C NMR (126 MHz, CDCl_3_) δ 171.23, 155.51, 134.06, 117.40, 79.99, 72.24, 69.94, 53.99, 52.48, 28.32. HPLC/MS MH^+^ 1601.1 (-Boc).

*Boc-Ser(O-allyl)-OH* (**iv**): To a solution of Boc-Ser(*O*-allyl)-OMe (1.24 g, 4.78 mmol) in a mixture of THF/MeOH/H_2_O (1:1:1, 75 mL) LiOH•H_2_O (502 mg, 11.96 mmol) was added and the reaction was stirred at room temperature until completion as determined by TLC. THF and MeOH were removed under reduced pressure and the resulting solution was partitioned between 1 N HCl (70 mL) and DCM (150 mL). The aqueous layer was extracted with DCM (×2) and the combined organics were dried over MgSO_4_, filtered, and concentrated under reduced pressure to afford the desired product, **iv** (1.1 g) as a colorless oil, which was used without further purification. ^1^H NMR (500 MHz, CDCl_3_) δ 5.85 (m, 1H), 5.40 (d, *J* = 7.9 Hz, 1H), 5.25 (m, 1H), 5.19 (q, *J* = 3.9 Hz, 1H), 4.45 (t, *J* = 3.9 Hz, 1H), 4.01 (d, *J* = 5.7 Hz, 2H), 3.90 (d, *J* = 4.5 Hz, 1H), 3.67 (q, *J* = 4.4 Hz, 1H).

*tert-butyl ((6S,9S,12S)-6-benzyl-9-(octanamidomethyl)-8,11-dioxo-4,14-dioxa-7,10-diazaheptadeca-1,16-dien-12-yl)carbamate (***v**): To a solution of the Fmoc-protected amine **iv** (1 eq.) and octanethiol (10 eq.) in THF (0.1 M) DBU (1.1 eq.) was added. The resultant solution was stirred at room temperature for 15 min and then concentrated under reduced pressure. The resultant residue was purified by column chromatography. The resultant residue (1.1 eq.), **iv** (1 eq.) and HATU (1.1 eq.) was suspended in DMF (0.3 M). To the suspension was added DIPEA (2.5 eq.) and the resulting solution was stirred at room temperature until completion, as determined by LCMS. Upon completion, the reaction mixture was diluted with EtOAc and washed with saturated aqueous NH_4_Cl (×2), water (×3), saturated aqueous NaHCO_3_ (×1), and brine (×1). The organics were dried over MgSO_4_, filtered, and concentrated under reduced pressure to afford **v** (195 mg). ^1^H NMR (500 MHz, CDCl_3_) δ 7.98 (d, *J* = 4.7 Hz, 1H), 7.28 (t, *J* = 3.8 Hz, 2H), 7.20 (q, *J* = 3.4 Hz, 3H), 7.08 (d, *J* = 8.6 Hz, 1H), 6.01 (s, 1H), 5.87 (m, 2H), 5.42 (d, *J* = 4.9 Hz, 1H), 5.28 (m, 1H), 5.24 (m, 1H), 5.19 (dd, *J* = 1.5, 10.5 Hz, 1H), 5.16 (dd, *J* = 1.5, 10.5 Hz, 1H), 4.40 (m, 1H), 4.35 (t, *J* = 3.8 Hz, 1H), 4.23 (t, *J* = 4.9 Hz, 1H), 3.98 (m, 4H), 3.78 (q, *J* = 4.8 Hz, 1H), 3.65 (q, *J* = 5.7 Hz, 1H), 3.58 (q, *J* = 4.9 Hz, 1H), 3.46 (m, 1H), 3.38 (m, 2H), 2.94 (q, *J* = 7.0 Hz, 1H), 2.77 (q, *J* = 7.2 Hz, 1H), 2.01 (q, *J* = 4.9 Hz, 1H), 1.64 (s, 6H), 1.56 (s, 1H), 1.46 (s, 9H), 1.26 (s, 8H), 0.87 (t, *J* = 6.9 Hz, 3H). ^13^C NMR (126 MHz, CDCl_3_) δ 175.55, 170.73, 134.72, 133.96, 129.31, 129.16, 128.37, 126.38, 117.78, 117.02, 72.29, 72.26, 72.16, 70.33, 69.43, 55.64, 54.95, 50.17, 41.78, 37.50, 36.29, 31.66, 29.22, 28.98, 28.34, 25.57, 22.61, 14.05. HPLC/MS MH^+^ 631.1.

*tert-butyl ((3S,6S,9S)-3-benzyl-6-(octanamidomethyl)-5,8-dioxo-1,11-dioxa-4,7-diazacyclopentadec-13-en-9-yl)carbamate (***vi**): The diene, **v** (158 mg, 0.25 mmol) was dissolved in CHCl_3_ (0.01 M) and sparged with argon for 10 min, and then a solution of Grubbs I (20 mol %) in CHCl_3_ (0.02 M) was added. The resultant solution was stirred under argon until completion as determined by LCMS. Column purification afforded **vi** (134 mg) as a mixture of alkene isomers. ^1^H NMR (500 MHz, CDCl_3_) δ 7.25 (m, 2H), 7.18 (m, 3H), 5.83 (m, 1H), 5.49 (dd, *J* = 8.5, 32.0 Hz, 1H), 5.20 (m, 1H), 4.33 (m, 2H), 4.12 (m, 1H), 3.97 (m, 2H), 3.85 (q, *J* = 5.7 Hz, 1H), 3.77 (m, 1H), 3.69 (q, *J* = 5.1 Hz, 1H), 3.57 (m, 2H), 3.44 (m, 1H), 3.31 (m, 2H), 2.91 (m, 1H), 2.76 (m, 1H), 2.25 (s, 1H), 2.13 (q, *J* = 7.2 Hz, 1H), 2.04 (m, 1H), 1.90 (d, *J* = 12.7 Hz, 3H), 1.82 (s, 5H), 1.71 (s, 2H), 1.56 (m, 2H), 1.50 (s, 1H), 1.42 (m, 11H), 1.23 (s, 13H), 0.85 (t, *J* = 4.1 Hz, 3H). ^13^C NMR (126 MHz, CDCl_3_) δ 176.40, 175.77, 175.58, 175.52, 171.65, 170.67, 170.57, 169.48, 169.24, 169.00, 168.77, 155.24, 155.18, 138.20, 129.37, 129.31, 129.15, 128.43, 128.42, 128.38, 128.34, 126.47, 126.44, 117.74, 72.23, 72.13, 71.19, 70.31, 70.22, 69.23, 69.46, 69.34, 69.02, 68.69, 67.29, 67.18, 56.44, 54.34, 50.95, 41.74, 37.45, 36.37, 36.32, 35.54, 35.06, 31.68, 31.66, 29.30, 29.27, 29.01, 28.33, 26.95, 26.86, 26.34, 26.32, 26.13, 22.61, 14.06. HPLC/MS MH^+^ 603.1.

*N-(((3S,6S,9S)-9-(2-aminoacetamido)-3-benzyl-5,8-dioxo-1,11-dioxa-4,7-diazacyclopentadec-13-en-6-yl)methyl)octanamide* (**9**): To a solution of **ii** (1 eq.) in CH_2_Cl_2_ (0.4 M) was added TFA (10% *v*/*v*). The resulting solution was stirred at room temperature until completion as determine by LCMS. The reaction mixture was concentrated under reduced pressure and the resultant TFA salt was used without further purification. The resultant residue (1.1 eq.), Boc-Gly-OH (1 eq.) and HATU (1.1 eq.) was suspended in DMF (0.3 M). DIPEA (2.5 eq.) was added to the suspension and the resulting solution was stirred at room temperature until completion, as determined by LCMS. Upon completion, the reaction mixture was diluted with EtOAc and washed with saturated aqueous NH_4_Cl (×2), water (×3), saturated aqueous NaHCO_3_ (×1), and brine (x1). The organics were dried over MgSO_4_, filtered, and concentrated under reduced pressure to afford Boc-**vi** (178 mg, 95%). To a solution of Boc-**vi**. (1 eq.) in CH_2_Cl_2_ (0.4 M), TFA (10% *v*/*v*) was added. The resulting solution was stirred at room temperature until completion, as determined by LCMSvto afford **9** (50 mg) which was purified by preparative HPLC to separate the alkene isomers. HRMS *m*/*z*: [M + H]^+^ Calculated for C_32_H_52_N_5_O_9_ 602.3918; Found 602.3871. ***Z*-9**: ^1^H NMR (500 MHz, MeOD) δ 8.35 (d, *J* = 7.3 Hz, 1H), 7.26 (d, *J* = 6.6 Hz, 3H), 7.18 (m, 2H), 5.79 (m, 2H), 4.46 (d, *J* = 2.3 Hz, 1H), 4.38 (m, 1H), 4.17 (d, *J* = 14.1 Hz, 1H), 4.03 (m, 3H), 3.83 (dd, *J* = 3.2, 9.8 Hz, 1H), 3.76 (dd, *J* = 8.1, 14.3 Hz, 1H), 3.54 (dd, *J* = 5.2, 10.5 Hz, 1H), 3.48 (q, *J* = 5.2 Hz, 1H), 3.35 (dd, *J* = 2.2, 10.5 Hz, 1H), 3.19 (dd, *J* = 2.4, 14.2 Hz, 1H), 2.92 (dd, *J* = 6.5, 13.0 Hz, 1H), 2.78 (dd, *J* = 8.7, 13.4 Hz, 1H), 2.20 (m, 2H), 1.93 (m, 2H), 1.85 (m, 2H), 1.75 (s, 1H), 1.59 (t, *J* = 6.7 Hz, 2H), 1.42 (d, *J* = 12.2 Hz, 2H), 1.31 (m, 11H), 0.88 (t, *J* = 6.7 Hz, 3H). ^13^C NMR (126 MHz, MeOD) δ 172.09, 170.08, 166.86, 138.15, 130.16, 129.01, 128.04, 127.50, 126.12, 69.47, 69.22, 69.02, 66.63, 57.29, 57.19, 55.21, 52.59, 41.30, 41.25, 40.60, 36.47, 35.67, 34.96, 34.47, 31.48, 29.01, 28.76, 26.41, 26.32, 25.82 (d, *J* = 2.9 Hz, 1C), 25.75, 25.65, 22.30. ***E*-9**: ^1^H NMR (500 MHz, DMSO) δ 7.35 (d, *J* = 8.4 Hz, 1H), 7.28 (t, *J* = 7.4 Hz, 2H), 7.24 (t, *J* = 4.1 Hz, 2H), 7.20 (m, 1H), 5.87 (m, 2H), 4.57 (t, *J* = 5.4 Hz, 1H), 4.35 (m, 2H), 4.10 (dd, *J* = 4.7, 12.2 Hz, 2H), 4.01 (dd, *J* = 5.7, 11.4 Hz, 1H), 3.94 (dd, *J* = 6.3, 12.7 Hz, 1H), 3.77 (m, 2H), 3.72 (s, 2H), 3.47 (m, 2H), 3.37 (dq, *J* = 3.2, 9.5 Hz, 2H), 2.92 (q, *J* = 6.5 Hz, 1H), 2.82 (q, *J* = 7.4 Hz, 1H), 2.19 (m, 2H), 1.89 (m, 1H), 1.59 (m, 2H), 1.42 (m, 1H), 1.31 (s, 9H), 0.89 (t, *J* = 6.9 Hz, 3H). HPLC/MS MH^+^ 560.3.

### 3.2. Preparation of Macrocycles ***17A*** and ***17B***

**Scheme 4:**
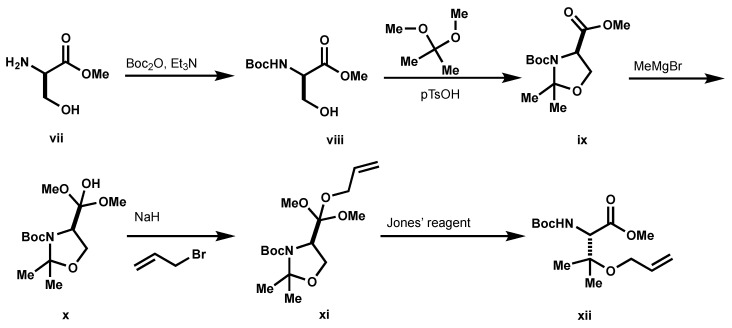
Synthesis of starting material towards **17A** and **17B** (see Materials and Methods for experimental detail).

*Boc-D-Ser-OMe* (**viii**, Scheme 4): To a solution of **vii** (1.0 g, 8.39 mmol) and Et_3_N (2.51 mL, 18.04 mmol) in THF (22 mL) at 0 °C, a solution of Boc_2_O was added (1.81 g, 8.31 mmol) in THF (7 mL) dropwise over 20 min. The resulting solution was warmed to room temperature, stirred at that temperature overnight, and then stirred at 50 °C for 3 h. The solvent was removed under reduced pressure and the resultant residue was taken up in ether (15 mL) and saturated aqueous NaHCO_3_ (20 mL). The aqueous phase was extracted with ether (×3). The organics were combined, dried over MgSO_4_, filtered, and concentrated under a reduced pressure to afford **viii** (1.61 g, 87%) as a colorless oil which was used without further purification. ^1^H NMR (500 MHz, CDCl_3_) δ 5.46 (s, 1H), 4.38 (s, 1H), 3.95 (m, 1H), 3.89 (m, 1H), 3.77 (s, 3H), 2.46 (t, *J* = 6.2 Hz, 1H), 1.44 (s, 9H).

*3-(tert-butyl) 4-methyl (R)-2,2-dimethyloxazolidine-3,4-dicarboxylate (***ix**): To a solution of **viii** (1.67 g, 7.33 mmol) in CH_2_Cl_2_ (9 mL) at 0 °C, 2,2-dimethoxypropane (4.50 mL, 36.66 mmol) and pTsOH (140 mg, 0.73 mmol) were added. The resulting mixture was warmed to room temperature and then stirred at room temperature overnight. The reaction mixture was then poured into saturated aqueous NaHCO_3_ (10 mL) and extracted with ether (×3). The organics were combined and washed with NaHCO_3_ and brine, dried over MgSO_4_, filtered, and concentrated under reduced pressure. The residue was purified by column chromatography using 1:1 hexanes: EtOAc as eluents to afford **ix** (1.63 g, 86%) as a colorless oil. ^1^H NMR (500 MHz, CDCl_3_) δ 4.48 (dd, *J* = 2.4, 6.8 Hz, 0.4H), 4.37 (dd, *J* = 3.0, 6.8 Hz, 0.6H), 4.13 (m, 1H), 4.03 (m, 1H), 3.75 (s, 3H), 1.66 (s, 1.89H), 1.63 (s, 1.42H), 1.52 (s, 1.77H), 1.49 (s, 5.11H), 1.40 (s, 5.29H).

*tert-butyl(R)-4-(2-hydroxypropan-2-yl)-2,2-dimethyloxazolidine-3-carboxylate* (**x**): To a solution of **ix** (1.63 g, 6.29 mmol) in THF (50 mL) at −20 °C MeMgBr (1.95 M in Et_2_O, 10.8 mL, 21.13 mmol) was added dropwise. The resulting mixture was stirred at 0 °C for 4 h. Saturated, aqueous NH_4_Cl was added to the reaction mixture to quench the reaction, and then extracted with EtOAc (×3). The organics were combined, washed with brine (×2), dried over MgSO_4_, filtered, and concentrated under reduced pressure. The residue was purified by column chromatography using 3:1 hexanes:EtOAc as eluents to afford **x** (982 mg, 60%). ^1^H NMR (500 MHz, CDCl_3_) δ 4.00 (m, 2H), 3.79 (s, 1H), 1.59 (s, 3H), 1.50 (s, 12H), 1.17 (d, *J* = 7.0 Hz, 6H). HPLC/MS MH^+^ 186.2 (-Boc + Na).

*tert-butyl (R)-4-(2-(allyloxy)propan-2-yl)-2,2-dimethyloxazolidine-3-carboxylate* (**xi**): To a solution of **x** (289 mg, 1.12 mmol) in DMF (5.2 mL) at 0 °C, NaH (60% in mineral oil, 90 mg, 2.24 mmol) was added, followed by allyl bromide (0.11 mL, 1.25 mmol). The resulting solution was warmed to room temperature, stirred for 1 h, and cooled back to 0 °C. The reaction was quenched with the addition of saturated aqueous NH_4_Cl and organics were extracted with EtOAc (×3). The organics were combined, washed with brine, dried over MgSO_4_, filtered, and concentrated under reduced pressure. The resultant residue was purified by column chromatography using 3:1 hexanes: EtOAc to afford **xi** (89 mg). ^1^H NMR (500 MHz, CDCl_3_) δ 5.88 (m, 1H), 5.25 (m,1H), 5.09 (d, 1H), 4.20 (d, *J* = 7.7 Hz, 1H), 3.94 (m, 2H), 3.87 (m, 1H), 1.60 (s, 3H), 1.49 (s, 3H), 1.48 (s, 9H), 1.22 (s, 3H), 1.17 (s, 3H). ^13^C NMR (126 MHz, CDCl_3_) δ 145.18, 136.00, 115.46, 80.16, 62.87, 29.71, 28.35. HPLC/MS MH^+^ 200.2 (-Boc).

*(S)-3-(allyloxy)-2-((tert-butoxycarbonyl)amino)-3-methylbutanoic acid* (**xii**): To a solution of **xi** (80 mg, 0.27 mmol) in acetone (3 mL), Jones’ reagent (2.5 M in H_2_O, 0.16 mL, 0.40 mmol) was added at 0 °C. The resulting mixture was warmed to room temperature, and then stirred at this temperature overnight. To the reaction mixture, celite (100 mg) and isopropanol (0.5 mL) were added, and the resulting precipitate was filtered through off through a plug of celite. The filtrate was adjusted to pH 9 with aqueous NaHCO_3_, and then concentrated under reduced pressure. The aqueous layer was washed with ether (×2) and acidified to pH 3 with citric acid. The resulting solution was extracted with EtOAc (×3) and the combined extracts were washed with brine (×2), dried over MgSO_4_, filtered, and concentrated under reduced pressure to afford **xii** (50 mg, 68%), which was used without further purification. ^1^H NMR (500 MHz, CDCl_3_) δ 5.89 (m, *J* = 5.5 Hz, 1H), 5.30 (q, *J* = 6.2 Hz, 1H), 5.20 (q, *J* = 3.8 Hz, 1H), 4.38 (s, 1H), 4.04 (m, 2H), 1.44 (s, 9H), 1.35 (s, 3H), 1.24 (s, 3H). ^13^C NMR (126 MHz, CDCl_3_) δ 172.36, 56.00, 133.90, 117.54, 80.37, 78.67, 63.60, 28.26, 22.64, 21.11.

*N-(((3S,6S,9S)-9-(2-aminoacetamido)-3-benzyl-10,10-dimethyl-5,8-dioxo-1,11-dioxa-4,7-diazacyclopentadec-13-en-6-yl)methyl)octanamide* (**17**): Macrocycles **17** were synthesized in the same manner as **9** with the modified residue substituted in where necessary. **Z-17**: ^1^H NMR (500 MHz, MeOD) δ 8.06 (q, *J* = 3.7 Hz, 1H), 7.64 (m, 1H), 7.54 (t, *J* = 4.0 Hz, 1H), 7.27 (t, *J* = 7.3 Hz, 2H), 7.23 (t, *J* = 4.1 Hz, 2H), 7.19 (m, 1H), 5.79 (m, 2H), 4.56 (s, 1H), 4.49 (m, 1H), 4.02 (m, 2H), 3.94 (t, *J* = 5.3 Hz, 2H), 3.90 (t, *J* = 3.8 Hz, 1H), 3.74 (q, *J* = 13.5 Hz, 2H), 3.62 (m, 1H), 3.45 (q, *J* = 4.2 Hz, 1H), 3.36 (q, *J* = 4.6 Hz, 1H), 3.34 (s, 1H), 2.97 (q, *J* = 7.1 Hz, 1H), 2.82 (q, *J* = 7.0 Hz, 1H), 2.14 (t, *J* = 7.6 Hz, 2H), 1.57 (m, 2H), 1.43 (s, 2H), 1.29 (m, 9H), 1.27 (s, 3H), 1.24 (s, 3H), 0.89 (t, *J* = 7.0 Hz, 3H). ^13^C NMR (126 MHz, MeOD) δ 175.69, 170.13, 169.79, 165.86, 138.42, 131.02, 128.93, 128.09, 127.22, 126.12, 77.02, 69.39, 67.88, 60.51, 60.33, 54.35, 53.18, 41.01, 40.15, 35.83, 35.70, 31.51, 29.00, 28.80, 25.47, 22.62, 22.31, 18.77, 13.02. **E-17**: ^1^H NMR (500 MHz, MeOD) δ 7.25 (m, 5H), 5.82 (m, 2H), 4.50 (s, 1H), 4.41 (t, *J* = 5.4 Hz, 1H), 4.24 (q, *J* = 5.6 Hz, 1H), 4.17 (q, *J* = 6.3 Hz, 1H), 4.05 (m, 2H), 3.96 (q, *J* = 5.5 Hz, 1H), 3.73 (d, *J* = 4.3 Hz, 2H), 3.52 (t, *J* = 4.7 Hz, 2H), 3.43 (q, *J* = 4.4 Hz, 1H), 3.34 (q, *J* = 4.2 Hz, 1H), 2.97 (m, 1H), 2.78 (q, *J* = 7.5 Hz, 1H), 2.18 (m, 2H), 1.60 (t, *J* = 7.2 Hz, 2H), 1.36 (s, 3H), 1.30 (m, 11H), 0.89 (t, *J* = 6.9 Hz, 3H). ^13^C NMR (126 MHz, MeOD) δ 176.54, 169.93, 138.18, 131.29, 129.02, 128.82, 128.13, 126.14, 77.43, 68.71, 65.98, 60.13, 58.53, 55.14, 51.81, 40.56, 36.52, 35.57, 31.49, 29.04, 28.84, 25.54, 22.47, 22.33, 21.13, 13.03. HPLC/MS MH^+^ 588.3.

### 3.3. Preparation of Heterocyclic Analog ***10***

*tert-butyl 4-((7-amino-5-methyl-[1,2,5]oxadiazolo[3,4-b]pyridin-6-yl)ethynyl)piperi-dine-1-carboxylate* (**21**): **18** and **19** were prepared using methods described in the literature [22,32]. A solution of **19** (1.35 g, 4.89 mmol), **20** (1.45 g, 7.15 mmol), (PPh_3_)_2_PdCl_2_ (687 mg, 0.98 mmol), and CuI (93 mg, 0.49 mmol) in Et_3_N (20 mL) was degassed with argon for 15 min and then heated at 80 °C for 24 h. The mixture was then stirred at room temperature for 2 days, diluted with EtOAc and filtered through a pad of celite. The filter cake was rinsed with EtOAc. The organics were washed with brine (×2), dried over MgSO_4_, filtered, and concentrated under a reduced pressure. The resultant residue was purified via column chromatography using hexanes:EtOAc as eluents to afford **21** (889 mg, 51%). ^1^H NMR (500 MHz, DMSO) δ 3.63 (m, 2H), 3.15 (s, 2H), 2.94 (m, 1H), 2.53 (s, 3H), 1.83 (m, 2H), 1.61 (m, 2H), 1.38 (s, 9H). HPLC/MS MH^+^ 358.3.

*tert-butyl (Z)-4-(2-(7-amino-5-methyl-[1,2,5]oxadiazolo[3,4-b]pyridin-6-yl)vinyl)piperidine-1-carboxylate* (**23**): To a solution of **21** (250 mg, 0.70 mmol) in EtOH (7 mL) under argon Pd(OH)_2_ was added (20% on carbon, 150 mg, 0.21 mmol). Argon was removed and reaction was conducted under an H_2_ atmosphere and stirred at room temperature. Upon completion, as determined by LCMS, the reaction mixture was diluted with EtOH and filtered through a pad of celite to remove the catalyst. The filter cake was rinsed with EtOH and the filtrate was concentrated under reduced pressure to afford **22** and **23** (276 mg crude) which were identified by HPLC/MS analysis. The crude mixture was used in the next step without further purification.

*(Z)-1-(4-(2-(7-amino-5-methyl-[1,2,5]oxadiazolo[3,4-b]pyridin-6-yl)vinyl)piperidin-1-yl)-2-butoxyethan-1-one (25) and 1-(4-(2-(7-amino-5-methyl-[1,2,5]oxadiazolo[3,4-b]pyridin-6-yl)ethyl)piperidin-1-yl)-2-butoxyethan-1-one* (**10**): To the crude mixture of **22** and **23** (252 mg, 0.70 mmol) in CH_2_Cl_2_ (16 mL) TFA was added (10% *v*/*v*, 1.61 mL, 21.0 mmol). The resulting mixture was stirred at room temperature until completion as determined by LCMS. Upon completion, the reaction mixture was concentrated under a reduced pressure to afford the TFA salts, which were immediately suspended with (0.70 mmol), n-butoxyacetic acid (0.10 mL, 0.77 mmol), and HATU (293 mg, 0.77 mmol) in DMF (3 mL). DIPEA (0.37 mL, 2.10 mmol) was added to the suspension, and the resulting mixture was stirred at room temperature until completion, as determined by LCMS. Upon completion, the reaction was diluted with EtOAc and washed with saturated, aqueous NH_4_Cl (×2), water (×2); saturated, aqueous NaHCO_3_ (×2); and brine (×2). The organics were dried over MgSO_4_, filtered, and concentrated under reduced pressure. The residue was purified by column chromatography using hexanes:EtOAc as eluents to afford **25** (300 mg) and **10** (10 mg) as yellow residues. **25**: ^1^H NMR (500 MHz, MeOD) δ 7.89 (s, 2H), 6.15 (d, *J* = 10.9 Hz, 1H), 5.87 (dd, *J* = 9.7, 10.7 Hz, 1H), 4.39 (d, *J* = 13.0 Hz, 1H), 4.12 (q, *J* = 18.1 Hz, 2H), 3.88 (s, 1H), 3.85 (d, *J* = 12.8 Hz, 1H), 3.51 (t, *J* = 6.6 Hz, 1H), 3.46 (q, *J* = 6.7 Hz, 2H), 2.98 (s, 1H), 2.94 (d, *J* = 12.0 Hz, 1H), 2.85 (d, *J* = 0.6 Hz, 1H), 2.80 (s, 1H), 2.58 (t, *J* = 11.7 Hz, 1H), 2.44 (s, 3H), 2.26 (m, 1H), 1.57 (m, 4H), 1.38 (m, 5H), 0.92 (t, *J* = 7.4 Hz, 3H). ^13^C NMR (126 MHz, MeOD) δ 169.65, 168.58, 158.30, 141.64, 140.78, 139.02, 120.46, 109.81, 78.08, 71.12, 70.88, 69.36, 44.37, 41.23, 37.48, 36.17, 31.33, 31.25, 23.91, 18.89, 12.79. HRMS m/z: [M + H]^+^ Calculated for C_19_H_28_N_5_O_3_ 374.2192; Found 374.2159. **10**: ^1^H NMR (500 MHz, CDCl_3_) δ 5.13 (br s, 2H), 4.62 (d, *J* = 13.2 Hz, 1H), 4.13 (d, *J* = 12.3 Hz, 2H), 4.00 (d, *J* = 13.6 Hz, 1H), 3.49 (q, *J* = 7.1 Hz, 2H), 3.03 (t, *J* = 12.0 Hz, 1H), 2.62 (s, 3H), 2.59 (d, *J* = 8.5 Hz, 1H), 1.87 (t, *J* = 12.8 Hz, 2H), 1.66 (m, 1H), 1.58 (m, 2H), 1.49 (m, 2H), 1.37 (m, 2H), 1.24 (s, 2H), 1.21 (s, 1H), 1.20 (s, 1H), 0.91 (t, *J* = 7.4 Hz, 3H). ^13^C NMR (126 MHz, CDCl_3_) δ 170.01, 167.86, 157.97, 139.23, 138.21, 114.14, 71.29, 70.72, 45.33, 42.13, 36.51, 34.10, 32.81, 31.67, 29.71, 25.37, 25.00, 24.02, 19.28, 13.88. HRMS *m*/*z*: [M + H]^+^ Calculated for C_19_H_30_N_5_O_3_ 376.2349; Found 376.2315.

### 3.4. Preparation of Heterocyclic Analog ***11***

*tert-butyl 4-(4-oxopentyl)piperidine-1-carboxylate* (**28**): To a flask containing CuCN (1.65 g, 18.42 mmol), dry Et_2_O (30 mL) was added. The resulting suspension was cooled to 0 °C and MeLi (1.6 M in Et_2_O, 23 mL, 36.85 mmol) was added dropwise. After addition, the resulting mixture was stirred at 0 °C for 5 min before the dropwise addition of a solution of **27** (1.00 g, 3.68 mmol) in Et_2_O (18.4 mL). The resulting reaction mixture was warmed to room temperature over an hour and then stirred at room temperature overnight. The reaction was quenched with saturated aqueous NH_4_Cl, and then extracted with CH_2_Cl_2_ (×2). The organics were dried over MgSO_4_, filtered, and concentrated under reduced pressure to afford **28** (703 mg, 71%) as a colorless oil. ^1^H NMR (500 MHz, CDCl_3_) δ 4.05 (d, *J* = 13.2 Hz, 2H), 2.65 (dt, *J* = 2.6, 12.8 Hz, 2H), 2.40 (t, *J* = 7.4 Hz, 2H), 2.12 (s, 3H), 1.63 (m, 2H), 1.58 (m, 2H), 1.44 (s, 9H), 1.36 (m, 1H), 1.21 (m, 2H), 1.05 (m, 2H). ^13^C NMR (126 MHz, CDCl_3_) δ 208.99, 154.90, 79.20, 43.99, 43.82, 35.98, 35.91, 32.07, 29.93, 28.48, 20.88. HPLC/MS MH^+^ 170.2 (-Boc).

*tert-butyl (S)-(1-oxo-1-(4-(4-oxopentyl)piperidin-1-yl)propan-2-yl)carbamate* (**29**): To a solution of **28** (200 mg, 0.74 mmol) in CH_2_Cl_2_ (10 mL) at 0 °C TFA was added (10% *v*/*v*, 1.14 mL, 14.85 mmol). The resulting solution was stirred at 0 °C until completion as determined by TLC. The reaction mixture was concentrated under reduced pressure and the resultant residue was immediately used. To a solution of the TFA salt (188 mg, 0.74 mmol) and Boc-Ala-OSu (203 mg, 0.71 mmol) in MeCN (3 mL) DIPEA was added (0.39 mL, 2.22 mmol) at 0 °C. The resulting mixture was stirred at 0 °C for 2 h, diluted with EtOAc and washed with saturated aqueous NH_4_Cl and H_2_O (×3). The organics were dried over MgSO_4_, filtered, and concentrated under reduced pressure. Residue was purified by column chromatography using 1:1 hexanes: EtOAc as eluents to afford **29** (122 mg, 50%) as a colorless oil. ^1^H NMR (500 MHz, CDCl_3_) δ 5.59 (s, 1H), 4.56 (m, 2H), 3.83 (t, *J* = 12.4 Hz, 1H), 3.00 (m, 1H), 2.57 (m, 1H), 2.42 (t, *J* = 6.7 Hz, 2H), 2.13 (s, 3H), 1.77 (m, 2H), 1.59 (t, *J* = 3.8 Hz, 2H), 1.49 (m, 2H), 1.43 (d, *J* = 4.0 Hz, 9H), 1.28 (dd, *J* = 6.9, 8.5 Hz, 1H), 1.10 (m, 3H).

*(S)-1-methyl-N-(1-oxo-1-(4-(4-oxopentyl)piperidin-1-yl)propan-2-yl)-1H-pyrazole-5-carboxamide* (**31**): To a solution of **29** (122 mg, 0.36 mmol) in CH_2_Cl_2_ (5 mL) at 0 °C, TFA was added (10% *v*/*v*, 0.55 mL, 7.16 mmol). The resulting mixture was stirred at 0 °C until completion as determined by TLC. The reaction mixture was concentrated under a reduced pressure and the resultant residue was used immediately without purification. The deprotected material (0.36 mmol) and **30** (80 mg, 0.36 mmol) were suspended in MeCN (1.5 mL) at 0 °C. DIPEA was added (0.19 mL, 1.08 mmol) to the suspension, and the resulting reaction mixture was stirred at 0 °C for 2 h and diluted with EtOAc. Organics were washed with saturated, aqueous NH_4_Cl and H_2_O (×3), dried over MgSO_4_, filtered, and concentrated under reduced pressure. The residue was purified by column chromatography using 1:1 hexanes: EtOAc as eluents to afford **31** (90 mg, 72%). (Rotational isomers present.) ^1^H NMR (500 MHz, CDCl_3_) δ 7.44 (d, *J* = 1.5 Hz, 1H), 7.28 (s, 1H), 6.60 (q, *J* = 2.6 Hz, 1H), 4.97 (q, *J* = 6.6 Hz, 1H), 4.56 (t, *J* = 12.9 Hz, 1H), 4.18 (s, 3H), 3.87 (m, 1H), 3.06 (m, 1H), 2.62 (m, 1H), 2.43 (q, *J* = 7.1 Hz, 2H), 2.13 (d, *J* = 3.7 Hz, 3H), 1.81 (m, 2H), 1.58 (m, 2H), 1.52 (m, 1H), 1.40 (q, *J* = 5.5 Hz, 3H), 1.24 (t, *J* = 18.2 Hz, 2H), 1.11 (m, 2H). ^13^C NMR (126 MHz, MeOD) δ 208.73, 170.13, 169.94, 158.81, 137.51, 106.76, 60.41, 45.90, 45.52, 45.41, 43.64 (d, *J* = 5.3 Hz, 1C), 42.85, 42.51, 39.26, 36.06, 35.89, 35.69, 35.62, 32.66, 32.47, 31.71 (d, *J* = 6.5 Hz, 1C), 29.98, 20.74, 19.42, 18.91, 14.21.

*(S)-N-(1-(4-(2-(7-amino-5-methyl-[1,2,5]oxadiazolo[3,4-b]pyridin-6-yl)ethyl)pip-eridin-1-yl)-1-oxopropan-2-yl)-1-methyl-1H-pyrazole-5-carboxamide (**11**) and (S)-N-(1-(4-(3-(7-amino-[1,2,5]oxadiazolo[3,4-b]pyridin-5-yl)propyl)pip-eridin-1-yl)-1-oxopropan-2-yl)-1-methyl-1H-pyrazole-5-carboxamide* (**35**): To a suspension of **31** (90 mg, 0.26 mmol) and **18** (28 mg, 0.26 mmol) in toluene (0.2M) tin(IV) chloride was added (2 eq). The resulting mixture was stirred at room temperature for 30 min, and then refluxed for 18 h. The reaction was cooled to room temperature and concentrated under reduced pressure. The resultant residue was dissolved in CH_2_Cl_2_ and washed with saturated aqueous NaHCO_3_ (×1). The aqueous layer was extracted with CH_2_Cl_2_ (×3). The organics were combined, washed with brine (×1), dried over MgSO_4_, filtered, and concentrated under reduced pressure. The residue was purified by column chromatography using 5% MeOH in CH_2_Cl_2_ as eluents to afford **11** and **35**. HRMS *m*/*z*: [M + H]^+^ Calculated for C_21_H_29_N_5_O_6_ 441.2363; Found 441.2313. **11**: ^1^H NMR (500 MHz, MeOD) δ 7.43 (d, *J* = 2.0 Hz, 1H), 6.83 (s, 1H), 4.97 (m, 1H), 4.47 (m, 1H), 4.06 (d, *J* = 5.6 Hz, 1H), 4.01 (d, *J* = 10.5 Hz, 1H), 3.12 (m, 1H), 2.65 (m, 1H), 2.46 (q, *J* = 7.0 Hz, 2H), 2.10 (d, *J* = 3.6 Hz, 3H), 1.78 (m, 2H), 1.55 (m, 3H), 1.36 (dd, *J* = 7.1, 13.9 Hz, 3H), 1.24 (m, 3H), 1.07 (m, 2H). ^13^C NMR (126 MHz, MeOD) δ 210.31, 170.8, 164.8, 137.12, 107.16, 45.69, 45.36, 45.22, 42.74, 42.55, 42.19, 37.73, 35.64, 35.43, 35.35, 35.23, 32.35, 32.21, 31.53, 31.43, 28.29, 20.32, 16.46, 15.98. **35**: ^1^H NMR (500 MHz, MeOD) δ 7.42 (q, *J* = 2.4 Hz, 1H), 6.83 (m, 1H), 6.29 (d, *J* = 4.5 Hz, 1H), 4.97 (m, 1H), 4.46 (m, 1H), 4.05 (s, 3H), 4.02 (s, 1H), 3.13 (m, 1H), 2.67 (m, 3H), 2.53 (d, *J* = 9.5 Hz, 1H), 1.88 (q, *J* = 12.7 Hz, 1H), 1.75 (m, 3H), 1.59 (s, 1H), 1.46 (q, *J* = 8.0 Hz, 1H), 1.35 (dd, *J* = 7.1, 13.0 Hz, 3H), 1.25 (s, 2H), 1.08 (m, 2H). ^13^C NMR (126 MHz, MeOD) δ 174.48, 159.60, 137.10, 107.15, 100.26, 45.23, 42.56, 38.87, 37.72, 35.53, 35.38, 32.20, 31.53, 31.43, 25.78, 23.05, 16.44, 15.95.

### 3.5. In Vitro Assay

pFastBac1-mouseGOAT and pGEX-GST-proGhrelin8His plasmid encoding for mouse proghrelin, fused to GST with TEV cleavage site to release proghrelin moiety, was a kind gift from the Brown and Goldstein laboratory [18]. The BL21Gold *E. coli* chemically competent cells were transformed with the pGEX-GST-proGhrelin8His plasmid and selected on agar plates with ampicillin. A few colonies were used to inoculate 50 mL LB, and cultures were grown overnight at 37 °C. The next morning, 10 mL of overnight culture was used to inoculate 1L LB. A total of 4 flasks with 1L LB each were inoculated with 10 mL of the overnight culture, and bacterial cultures were grown at 37 °C until they reached OD_600_ ~ 0.6. The cultures were chilled on ice for 30 min and then 0.25 mM of IPTG was added to each culture. The cultures were then moved back to a shaker set to 18 °C and incubated overnight. Cells were harvested and resuspended in 200 mL buffer containing 25 mM Tris-HCl pH 8.0, 150 mM NaCl, 1 mM EDTA and 0.5 mg/mL lysozyme. The cell suspension was sonicated, and cell debris was removed by centrifugation. The soluble fraction was passed through glutathione resin by gravity. The resin was washed with 75 mL of lysis buffer without lysozyme. The GST-proGhrelin8His protein was eluted with lysis buffer without lysozyme, supplemented with 15 mM reduced glutathione, GSH. About 50 mg of target protein, as evaluated by SDS-PAGE, was eluted. The eluate was supplemented with 1 mM DTT and about 7.5 mg of recombinant GST-TEV protease (see below) was added. The solution was incubated at 16 °C overnight to allow complete release of proghrelin8His from GST. Prior to Ni-NTA chromatography, 5 mM CaCl_2_ was added, and the solution was spun down at 4000× *g* for 10 min. After elution from Ni-NTA resin, protein solution was dialyzed against buffer containing 10 mM Tris-HCl pH 8.5, 50 mM NaCl, 10% glycerol and 0.01% CHAPS using 3 kDa cutoff membrane with 3 buffer exchanges. The protein was quantified by absorbance at A_280_ and qualified by SDS-PAGE.

*Generation of GST-TEV protease*. The pMHT vector [34] (gift from Dr. Arbing, UCLA) was linearized by PCR reaction with primers outside of MBP gene: forw- 5′ gcgaccatcctccaaaatcgggagaaagcttgtttaaggggccg 3′ and rev- 5′ caataacctagtataggggacatggttaatttctcctctttaatg 3′. The resulting linear plasmid was used to clone GST gene in-frame at 5′ of TEV gene by in vitro Gibson assembly. The GST gene was prepared by PCR amplification with primers: form 5′ atgtcccctatactaggttattg and rev 5′ cgattttggaggatggtcgc 3′ from pGEX-GST-proGhrelin8His plasmid as a template. The final bacterial expression vector, pGST-TEV was used for expression and purification of GST-TEV fusion protein. BL21Gold chemically competent *E. coli* cells were transformed with pGST-TEV plasmid and entire transformation mixture was used to inoculate 50 mL LB supplemented with kanamycin. Next day, 10 mL of the overnight culture was used to inoculate 1 L of LB. Four flasks with 1 L LB each were inoculated with 10 mL of the overnight culture, and bacterial cultures were grown at 37 °C till OD600 ~ 0.8. was reached. After adding 1 mM IPTG, culture was allowed to grow for 4 h, and cells were harvested by centrifugation. The cell pellet was resuspended in 200 mL lysis buffer containing 25 mM Tris-HCl pH 8.0, 150 mM NaCl, 1 mM EDTA, 5% glycerol, 0.5 mg/mL lysozyme and 1 mM DTT. Cell suspension after freeze–thaw cycle was sonicated and cellular debris was removed by centrifugation. The soluble fraction was bound to glutathione resin by gravity. The resin was washed with 50 mL washing buffer (lysis buffer without lysozyme and DTT) and fusion GST-TEV protein was eluted with 25 mL washing buffer supplemented with 15 mM reduced glutathione (GSH). The elution was fractionated into 5 fractions and after SDS-PAGE/Coomassie analysis, fractions containing most of the GST-TEV protein were combined and dialyzed against buffer containing 25 mM Tris-HCl pH 8.0, 150 mM NaCl and 50% glycerol using 3 kDa cutoff membrane with 3 buffer exchanges. The total yield was roughly 8 mg per 1 L bacterial culture.

*The baculovirus expression of mouse GOAT*. DH10Bac *E. coli* strain (ThermoFisher, Waltham, MA, USA) was transformed with pFastBac1-mouseGOAT plasmid, and Bacmid DNA from 12 white colonies was analyzed by DNA sequencing and PCR reaction. All clones were transfected to sf9 cells and level of mGOAT expression was compared between clones from cells producing P2. The clone with the highest mGOAT expression level was used to produce P3. For membrane isolation, sf9 cells were seeded at 2–4 × 10^6^ /mL in total 1 L volume of sf-900 serum-free medium (ThermoFisher) and infected with P3 virus. At day 2 post-infection, cells were harvested and resuspended in 40 mL of buffer containing 50 mM NaPi pH 7.2, 150 mM NaCl, 1 mM EDTA, 100 μM bis (4-nitrophenyl) phosphate, 2.5 μg/mL aprotinin, 10 μg/mL leupeptin, 10 μg/mL pepstatin A. Cell suspension was briefly sonicated and cell debris was removed by centrifugation at 3000× *g* for 10 min. Membrane fraction was collected from supernatant by centrifugation at 100,000× *g* for 1 h. Membrane pellet was resuspended in storage buffer (50 mM NaPi pH 7.2, 150 mM NaCl and 10% glycerol) and kept at −80 °C. *Acyltransferase assay*: The assay conditions included per 50 μL reaction: 50 μg of total sf9 membranes, various (for modality experiment) or fixed (for IC_50_) concentrations of recombinant proghrelin8His peptide and various concentrations of tested compound (for IC_50_), 100 μM palmitoyl CoA, 50 mM HEPES pH 7.0 and 1 μM [^3^H] octanoyl CoA (~5.5 dpm/fmol—American Radioactive Chemicals). After incubation of the reaction mixture at 37C for 10 min, tubes were placed on ice and 10 μL of 1M HCl was added to each tube. Following the addition of 740 μL of cold quench buffer (50 mM NaPi pH 7.4, 10 mM Imidazole, 150 mM NaCl, 100 μM bis (4-nitrophenyl) phosphate, 1 mM phenylmethylsulfonyl fluoride and 0.1% Triton), 0.2 mL of 50% Ni-NTA slurry was added to each reaction. Tubes were incubated at 4 °C for 1 h with rotation to capture proGhrl8His. After washing Ni resin with 40 mM Imidazole, all bound proghrelin8His was eluted with 250 mM Imidazole, and an amount of octanylated proghrelin was assessed with scintillation counting.

### 3.6. INS1-Cellular Assay

*Generation of Recombinant Retrovirus for mGOAT Expression.* The mouse GOAT cDNA with C-terminal HA tag was amplified from pcDNA3.1-mouseGOAT-HA vector (gift from Brown and Goldstein lab [2]) using the following primers: mGOAT_attb1 (forward primer) 5′-gggg*acaagtttgtacaaaaaagcaggct*acc**atgg****attggctccagctc**—3′(*attb1* recombination site is in italics, 5′ of mGOAT coding sequence is in **bold**, and Kozak coding sequence is underlined) and mGOAT_attb2 (reverse primer) 5′-gggg*accactttgtacaagaaagctgggt***ctaagcgtaatctggaacatc** -3′ (attb2 recombination site is in *italics*, 3′ of mGAOT-HA coding sequence is in **bold**, and stop codon is underlined). The PCR product was cloned into donor vector pDONR221 (ThermoFisher) with BP clonase according to manufacturer’s instructions. Positive clones were verified by DNA sequencing with M13F and M13R primers. The resulting entry plasmid pDONR221-mGOAT-HA was used to transfer mGOAT-HA cDNA to destination vector pBabe-puro (Addgene cat# 51070 [35]) using LR clonase according to manufacturer’s instructions. Positive clones were verified by DNA sequencing with pBABE-5 and pBABE-3 primers. The resulting plasmid, pBabe-puro-mGOAT-HA, was used to generate retrovirus. The retrovirus was packed in 293T PhoE (Phoenix-ECO AT0CC^®^ CRL-3214^™^) cells and used to infect INS-1 cells. The stable INS/GOAT cells were selecting on 1 μg/mL puromycin and GOAT expression was confirmed with immunoblot of membrane fraction isolated from antibiotic resistant culture using anti-HA antibody.

*Generation of Lentivirus for Expression of Ghrelin*. The cDNA for mouse preproghrelin was subcloned from pcDNA3.1-preproghrelin (gift from Brown and Goldstein lab [18]) into pULTRA vector (Addgene cat# 24129 [36]) with *XbaI* and *BamHI* restriction enzymes. Using of these restriction sites for cloning will result in the creation of bi-cistronic expression of ghrelin along with EGFP to facilitate identification of positive cells by fluorescence. The above restriction sites were engineered via PCR reaction using the following primers: ghrl_pultra_forw 5′-taccgagctc*tctaga***atgctgtcttcaggc**-3′ (5′ of preproghrelin coding sequence is in **bold**; *XbaI* site is in *italics*) and ghrl_pultra_rev 5′-agcggccgc*ggatcc***ttacttgtcagctggc** -3′ (3′ of preproghrelin coding sequence is in **bold**, stop codon is *underlined*, and *BamHI* site is *italics*). The recombinant lentivirus encoding preproghrelin cDNA was packed in Lenti-X 293T cells (Takara cat# 632180) by co-transfection of pUltra-EGFP-mouse-preproghrelin, with packaging encoding plasmid pCMV ΔR8.2 (Addgene cat# 12263) and envelope encoding plasmid pCMV-VSV g (Addgene cat# 8454). The INS/GOAT cells were infected with recombinant lentivirus to generate INS/GOAT/GHRL cell line. After few passages, the population of cells with puromycin resistance and ~90% fluorescence was saved for cell-based assay.

*Cellular assay*. INS/GOAT/GHRL cell line was routinely cultured in RPMI medium supplemented with 10% FBS, 1% Pen/Strep, 10 mM HEPES pH 7.2 and 50 μM 2-mercaptoethanol. Day 0: One 10 cm dish at full confluency was used to seed one 96-well plate. Day 1: Growth media was removed and cells were washed with PBS prior to adding fresh growth media as above but without 2-mercaptoethanol. The serial dilutions of tested compound at 50× concentration were prepared in vehicle containing growth media without 2-mercaptoethnol supplemented with 6% DMSO and were added to cells in duplicates. Day 2: 10 μL of growth media were removed from each well and amount of secreted acyl-ghrelin was measured with ELISA (Cayman cat# 10006307).

## 4. Conclusions

Ghrelin signaling continues to be an active area of basic biology research and drug discovery. The discovery of GOAT and its role in maturing ghrelin in the stomach provided an opportunity for pharmacological intervention without accessing the central nervous system. These efforts were largely empirical due to a lack of structural data for the system. However, we have learned a great deal. Peptidomimetic inhibitors that compete for substrate binding can perform well in vitro but, thus far, their utility in cell culture and in animals has been limited. The high-throughput screens developed in the private identified compounds uniformly appear to complete for binding at the co-enzyme site. Several of these molecules advanced through pre-clinical developments and into human trials as therapy for diabetes type II, Prader-Willi syndrome and alcohol use disorder. We developed a six-step synthesis of heterocyclic GOAT inhibitors based on a hybrid design that utilizes a piperidinyl ethyl linker and a late-stage oxadiazolopyridine, forming annulation. The potent activity of novel product **11** both in vitro and in cells indicates this approach has the potential to identify new GOAT inhibitors with increasingly refined properties.

## 5. Patents

Patrick G. Harran and Emily Murzinski (2020). Compositions and Methods for Treating Obesity. Provisional Patent Application 62/979,915. *Filed 21 February 2020;* Patrick G. Harran, Peter Tontonoz, David Strugatsky, Hui Ding, and Emily Murzinski (2017). Inhibitors of Ghrelin *O*-Acyl Transferase. Provisional Patent Application. *Filed 20 December 2017.*

## Data Availability

Not available.
